# Retrospective Study Demonstrating High Rates of Sustained Virologic Response After Treatment With Direct-Acting Antivirals Among American Indian/Alaskan Natives

**DOI:** 10.1093/ofid/ofz128

**Published:** 2019-07-04

**Authors:** Jorge Mera, Kartik Joshi, Karla Thornton, Terry Box, John Scott, Miranda Sedillo, Paulina Deming, Crystal David, Whitney Essex, Richard Manch, Anita Kohli

**Affiliations:** 1 Division of Infectious Diseases, Cherokee Nation W.W. Hastings Hospital, Tahlequah, Oklahoma; 2 Division of Hepatology, Creighton University School of Medicine, St. Joseph’s Hospital and Medical Center, Phoenix, Arizona; 3 Division of Infectious Diseases, Project ECHO, University of New Mexico Health Sciences Center, University of New Mexico, Albuquerque, New Mexico; 4 Division of Gastroenterology, University of Utah School of Medicine, Salt Lake City, Utah; 5 Division of Allergy and Infectious Diseases, University of Washington School of Medicine, Seattle, Washington; 6 Institute for Liver Health, Chandler, Arizona; 7 Division of Infectious Disease, Creighton University School of Medicine, St. Joseph’s Hospital and Medical Center, Phoenix, Arizona

**Keywords:** AI/AN, HCV, direct-acting antivirals

## Abstract

**Background:**

Treatment for chronic hepatitis C virus (HCV) has rapidly evolved to simple, well-tolerated, all-oral regimens of direct-acting antivirals (DAAs). There are few data on the epidemiology of HCV in American Indians/Alaska Natives (AI/ANs), a population disproportionately affected by HCV.

**Methods:**

In this retrospective cohort study, all HCV-infected AI/AN patients treated with DAA therapies between January 1, 2014, and February 24, 2016, in specialty clinics or by primary care clinicians participating in Extension for Community Healthcare Outcomes (ECHO) were included. Demographic, clinical, and virologic data on all patients treated for HCV from pretreatment through sustained virologic response at 12 weeks (SVR12) were collected.

**Results:**

Two hundred eighty patients were included; 71.1% of patients (n = 199) were infected with genotype 1 (GT1), 18.2% (n = 51) with GT2, and 10.7% with (n = 30) GT3. At baseline, 26.1% (n = 73) patients had cirrhosis and 22.6% (n = 56) had active substance use disorder; eighty-eight percent (n = 232) of patients achieved SVR12. Among the 165 GT1 patients treated with sofosbuvir (SOF)/ledipasvir for 8, 12, and 24 weeks, SVR12 was achieved by 91.5% (n = 54), 92.2% (n = 71), and 100% (n = 13), respectively. Among GT2 patients, 87.2% (n = 34) and 71.4% (n = 5) treated with 12 and 16 weeks of SOF/ribavirin (RBV) achieved SVR12, respectively. Among GT3 patients, 100% (n = 2) and 83.3% (n = 20) treated with 12 and 24 weeks of SOF/RBV achieved SVR12, respectively. SVR12 rates remained high among patients with active substance use disorder.

**Conclusions:**

DAA therapies are highly efficacious in HCV-infected AI/ANs. SVR12 rates remained high among patients with active substance use disorder. More steps must be taken to increase access to treatment for this underserved, vulnerable population.

Globally, an estimated 71 million people are infected with chronic hepatitis C virus (HCV) [1]. According to the most recent National Health and Nutrition Examination Survey (NHANES) study, in the United States, approximately 2.7 million people are infected with HCV [2]. This estimate, however, fails to account for highly vulnerable populations, including the homeless, the incarcerated, the active members of the military, and the American Indians and Alaskan Natives (AI/ANs) living on reservations. When these critical populations are factored into consideration, that prevalence of HCV in the United States may be as high as 5.2 million persons [3, 4].

In the United States, AI/ANs appear to be disproportionately affected by the HCV epidemic, with as much as a 4- to 5-fold higher prevalence of HCV (up to 8.6%) compared with other racial groups including Caucasians and Hispanics patients [5, 6]. Similarly, AI/ANs have higher rates of acute HCV compared with Caucasians, and are they more likely to experience chronic liver disease–related and specifically HCV-induced mortality [7–10].

Although direct-acting antivirals have shifted therapy for chronic HCV from complex, poorly tolerated regimens with low effectiveness, to simple, well-tolerated, highly effective, all-oral regimens [11–13], data on the effectiveness of DAAs in AI/ANs remain limited [14–16]. The primary aims of this study were to determine the effectiveness of DAA regimens in an AI/AN population infected with chronic HCV and to provide the first large, detailed analysis of the demographics, clinical characteristics, risk factors, and comorbidities of HCV-infected AI/ANs treated with DAAs.

## METHODS

### Study Population and Antiviral Regimens

All AI/AN patients with chronic HCV treated through Project Extension for Community Healthcare Outcomes (Project ECHO) between January 1, 2014, and February 24, 2016, were identified. Project ECHO is a collaborative teleconferencing mechanism designed to increase access of specialty care (eg, HIV, HCV) to underserved populations by pairing experienced clinician mentors from ECHO hubs with providers in underserved areas; these providers are provided with mentorship and modules to foment continuous learning, enabling them to treat patients with complex specialty diseases [17].

Participating ECHO hubs included the Cherokee Nation Health Services Hospital and Clinics in North Eastern Oklahoma; St. Joseph’s Hospital and Medical Center, Phoenix, Arizona; Harborview Medical Center, Seattle, Washington; the University of New Mexico, Albuquerque, New Mexico; and the University of Utah, Salt Lake City, Utah. Most participating providers treating HCV were located in areas where subspecialists were not available to treat HCV, and they were federally qualified health care centers or tribal/Indian Health Service clinics. DAA treatment regimens were chosen based on the current American Association for the Study of Liver Diseases (AASLD) guidelines at the time of treatment initiation and included sofosbuvir (SOF), ledipasvir (LDV), simeprevir (SIM), ombitasvir (OBV), paritaprevir (PTV), ritonavir (RTV), dasabuvir (DSV), ribavirin (RBV), and/or pegylated interferon (PEG) [18].

Patients were included if they (a) were ≥18 years old and (b) had completed a DAA-based HCV treatment regimen. Patients who did not follow-up 12 weeks after treatment completion were characterized as missing.

Data on DAA regimens with which fewer than 2 patients were treated were excluded.

### Variables

Demographics and other baseline variables were collected using electronic medical records, along with manual chart reviews. All data were systematically abstracted, de-identified, and placed into a standardized electronic database. Baseline laboratory values used for analysis were taken within 6 months before treatment initiation, including HCV genotype, HCV viral load (pretreatment and sustained virologic response at 12 weeks [SVR12]), alanine aminotransferase (ALT), aspartate aminotransferase (AST), platelet count, creatinine, total bilirubin, international normalized ratio (INR), and albumin. Clinical characteristics included prior and current HCV treatment regimens, duration of therapy, and coinfections with HIV and hepatitis B virus (HBV). Stage of liver fibrosis was performed at each institution according to their protocols, which included 1 or more of the following methods: Fibrosure, AST to platelet ratio index (APRI), FIB-4 index, liver biopsy, and imaging using computed tomography (CT), magnetic resonance imaging (MRI), vibration-controlled transient elastography (Fibroscan), or ultrasound.

A patient was characterized as having cirrhosis if they fulfilled any of the following criteria: (1) liver biopsy with Metavir 4 or Ishak 5–6 stage fibrosis; (2) the presence or history of 1 or more of the following: ascites, spontaneous bacterial peritonitis (SBP), esophageal varices, or hepatic encephalopathy; (3) the presence of 2 or more laboratory or imaging indicators: *laboratory:* (A) Fibrosure/Fibrotest >0.75, (B) Fib-4 index >3.25, (C) APRI >2, (D) platelets <140 000/mL; *imaging*: (A) transient elastography (Fibroscan) >12.5 kPa, (B) imaging revealing signs of portal hypertension: spleen size >13 cm, portal flow mean velocity <12 cm/sec, portal vein diameter >13 mm. Decompensated cirrhosis was defined as the presence or history of ascites, variceal hemorrhage, spontaneous bacterial peritonitis, hepatocellular carcinoma (HCC), hepatorenal syndrome, hepatopulmonary syndrome, or hepatic encephalopathy.

Additional data were gathered on a subset of 249 patients including the presence of concomitant diabetes mellitus (DM) and hemoglobin A1c (HbA1c); psychiatric disease (anxiety, post-traumatic stress disorder [PTSD], bipolar, mood disorder, depression, schizophrenia); tobacco use; alcohol use disorder; and risk factors for HCV transmission (intravenous drug use [IVDU], intranasal drug use, tattoos, history of blood transfusions, sexual intercourse with HCV-infected people, health care worker, HCV-infected mother, history of sharing personal items (eg, razors). Within this subset, the presence or absence of active substance use disorder at the time of HCV treatment was determined by reviewing 248 medical records. For 98.8% (n = 245) of patients, active substance use disorder was assessed by a physician both before HCV treatment evaluation and during treatment. Additionally, 67% (n = 167) of patients had a urine drug screen (UDS) performed at the time of HCV treatment evaluation and/or during treatment. A patient was characterized as having active substance use disorder if they had a positive UDS test result for a substance that was not prescribed or if it was documented by the medical provider. Two-hundred eleven of these patients had a pretreatment abdominal ultrasound performed to screen for steatosis and/or the presence of advanced liver disease and liver cancer. Based on a positive ultrasound and completion of a questionnaire assessing alcohol use disorder and/or physician interview, steatosis was ascribed to alcoholic liver disease or nonalcoholic fatty liver disease (NAFLD).

This study was approved by the institutional review boards of the Cherokee Nation W.W. Hastings Hospital, the University of Washington, the University of New Mexico, the University of Utah, and St. Joseph’s Hospital and Medical Center, Phoenix, Arizona.

### Statistical Analysis

Continuous variables were reported as mean ± SD. Categorical variables were reported by frequency in the designated population and percentage. Differences in continuous and categorical variables were assessed using *t* tests and χ^2^ analysis, respectively. *P* values of less than .05 were deemed statistically significant. All statistical analysis was completed using GraphPad Prism 6 (GraphPad Software, San Diego, CA; www.graphpad.com).

## RESULTS

A total of 284 HCV-infected AI/AN patients were identified. Four patients were excluded because they received a regimen that fewer than 2 patients received. In all, 280 patients were included and eligible for analysis.

### Baseline Characteristics

The majority of patients were infected with HCV genotype 1 (GT1; n = 199, 71.1%) with genotype 1a being the most common subtype (n = 164; 58.6%), followed by genotype 1b (n = 27, 9.6%). Eighteen percent (n = 51) of the population was infected with HCV genotype 2 (GT2), and 10.7% (n = 30) of patients were infected with HCV genotype 3 (GT3). The average body mass index (BMI) in the aggregate study population was 30.6 kg/m^2^.

Twenty-six percent of the population (n = 73) had cirrhosis (Table 1). Among patients with cirrhosis, 31.5% (n = 23) had a history of decompensated cirrhosis. Two patients (0.7%), 1 with GT1 and 1 with GT3, were found to have HCC. Two and 3 HCV GT1 patients were coinfected with HIV and HBV, respectively. Ninety-four patients (37.8%) had a reported history of alcohol use disorder that may have contributed to liver disease (Table 1).

**Table 1.** Demographics and Clinical Characteristics of 280 HCV-Infected American Indian/Alaska Natives

	Total	GT1										GT2		GT3	
		SOF/LDV			SOF/LDV/ RBV	SOF/RBV		SOF/PEG/ RBV	SIM/ SOF	SIM/SOF/ RBV	OBV/PTV/ DSV/r ± RBV	SOF/RBV		SOF/RBV	
Duration, wk		8	12	24	12	8	24	12	12	12	12	12	16	12	24
No.	280	69 (24.6)	83 (29.6)	13 (4.6)	8 (2.9)	2 (0.7)	2 (0.7)	6 (2.1)	6 (2.1)	5 (1.8)	5 (1.8)	44 (15.7)	7 (2.5)	2 (0.7)	28 (10)
Age, y															
19–30	10 (3.6)	6 (8.7)	1 (1.2)	―	―	―	―	―	―	―	―	1 (2.3)	―	―	2 (7.1)
31–40	38 (13.6)	18 (26.1)	7 (8.4)	1 (7.7)	―	―	1 (50)	―	―	―	2 (40)	6 (13.7)	―	―	3 (10.7)
41–50	54 (19.3)	13 (18.8)	16 (19.3)	2 (15.4)	3 (37.5)	―	―	2 (33.3)	―	―	―	7 (15.9)	2 (28.6)	―	9 (32.1)
51–60	118 (42.1)	22 (31.9)	39 (47.0)	4 (30.8)	4 (50)	1 (50)	1 (50)	2 (33.3)	4 (66.7)	3 (60)	1 (20)	21 (47.7)	2 (28.6)	2 (100)	12 (42.3)
61–70	53 (18.9)	8 (11.6)	18 (21.7)	5 (38.5)	1 (12.5)	1 (50)	―	2 (33.3)	2 (33.3)	2 (40)	2 (40)	9 (20.5)	2 (28.6)	―	1 (3.6)
71–80	7 (2.5)	2 (2.9)	2 (2.4)	1 (7.7)	―	―	―	―	―	―	―	―	1 (14.3)	―	1 (3.6)
BMI, kg/m^2^	30.6 ± 7.8	30.9 ± 9.6	30.9 ± 7.3	31.0 ± 6.4	33.2 ± 8.9	41.6 ± 15.5	24.3 ± 0.6	27.6 ± 5.8	28.4 ± 7.0	29.1 ± 5.8	31.8 ± 4.7	30.2 ± 7.9	31.6 ± 6.7	26.1 ± 0.6	29.6 ± 6.2
HCV genotype															
1^c^	8 (2.9)	4 (5.8)	4 (4.8)	―	―	―	―	―	―	―	―	―	―	―	―
1a	164 (58.6)	56 (81.1)	70 (84.3)	12 (92)	6 (75)	1 (50)	2 (100)	6 (100)	5 (83.3)	5 (100)	1 (20)	―	―	―	―
1b	27 (9.6)	9 (13.0)	9 (10.8)	1 (8)	2 (24)	1 (50)	―	―	1 (16.7)	―	4 (80)	―	―	―	―
2^c^	51 (18.2)	―	―	―	―	―	―	―	―	―	―	44 (100)	7 (100)	―	―
3	30 (11.4)	―	―	―	―	―	―	―	―	―	―	―	―	2 (100)	28 (100)
Other liver disease															
HIV/HCV	2 (0.7)	1 (1.4)	―	―	―	―	―	―	1 (16.7)	―	―	―	―	―	―
HBV/HCV	3 (1.0)	―	3 (3.6)	―	―	―	―	―	―	―	―	―	―	―	―
HCC	2 (0.7)	―	1 (1.2)	―	―	―	―	―	―	―	―	―	―	―	1 (3.6)
Cirrhosis^a^	73 (26.1)	2 (2.9)	28 (33.7)	9 (69.2)	5 (62.5)	―	1 (50)	2 (33.3)	2 (33.3)	4 (80)	―	7 (15.9)	4 (57.1)	―	9 (32.1)
APRI ≥ 2^b^	48 (17.1)	2 (2.9)	14 (16.9)	6 (46.2)	3 (37.5)	―	2 (100)	1 (16.7)	1 (16.7)	1 (20)	―	7 (15.9)	2 (28.6)	―	9 (32.1)
Decomp. cirrhosis	23 (8.2)	―	11 (13.3)	1 (7.7)	2 (25)	1 (50)	―	―	―	1 (20)	―	3 (6.8)	1 (14.3)	―	3 (10.7)
Treatment experienced	36 (12.9)	1 (1.4)	12 (14.5)	9 (69.2)	1 (12.5)	―	―	1 (16.7)	2 (33.3)	1 (20)	―	5 (11.4)	2 (28.6)	―	2 (7.1)
IFN/RBV	19 (52.8)	1 (100)	6 (50.0)	6 (66.7)	―	―	―	―	2 (100)	―	―	3 (60)	―	―	1 (50)
DAA, IFN/RBV	6 (16.7)	―	2 (16.7)	1 (11.1)	―	―	―	1 (100)	―	1 (100)	―	―	―	―	1 (50)
Other	11 (30.1)	―	4 (33.3)	―	―	―	―	―	―	―	―	2 (40)	2 (100)	―	―
Baseline labs, mean ± SD															
HCV RNA (106), IU/mL	3.0 ± 4.4	1.6 ± 2.7	3.2 ± 4.1	2.8 ± 2.3	3.8 ± 5.7	0.7 ± 1.0	3.4 ± 2.7	3.0 ± 3.4	2.0 ± 1.6	1.1 ± 1.0	1.7 ± 2.0	5.0 ± 5.5	2.1 ± 2.6	1.9 ± 0.4	4.4 ± 7.4
ALT	88.1 ± 76.3	70.9 ± 54.0	84.0 ± 53.0	118.2 ± 82.8	80.0 ± 60.3	57.5 ± 38.9	129.5 ± 120.9	93.3 ± 56.7	78.8 ± 59.7	126.4 ± 75.6	112.0 ± 68.4	80.0 ± 66.0	77.1 ± 60.6	76.0 ± 49.5	136.8 ± 157.0
AST	67.9 ± 53.5	52.0 ± 30.1	68.5 ± 36.5	125.4 ± 121.6	61.9 ± 28.5	52 ± 35.4	54.5 ± 0.71	61.3 ± 33.4	44.5 ± 23.8	98 ± 46.7	75.8 ± 33.9	61.8 ± 51.6	70.6 ± 45.3	76.0 ± 53.7	91.0 ± 89.0
Platelets	217.6 ± 87.7	257.6 ± 63.8	215.8 ± 102.5	144.7 ± 78.5	147.5 ± 75.2	287.5 ± 55.9	214.5 ± 105.4	221.7 ± 51.3	193.3 ± 56.8	135.4 ± 27.5	254.4 ± 101.7	220.8 ± 81.5	123.3 ± 41.6	276.5 ± 61.5	206.9 ± 82.8
Creatinine	0.9 ± 0.2	0.9 ± 0.2	0.9 ± 0.2	0.8 ± 0.1	0.8 ± 0.3	0.81 ± 0.3	1.05 ± 0.07	1.0 ± 0.3	0.95 ± 0.18	1.0 ± 0.26	0.7 ± 0.1	0.9 ± 0.3	1.0 ± 0.2	0.7 ± 0.2	0.9 ± 0.2
Bilirubin	0.7 ± 0.6	0.6 ± 0.3	0.7 ± 0.4	1.5 ± 1.9	1.2 ± 0.8	0.4 ± 0.14	0.65 ± 0.21	0.6 ± 0.2	0.45 ± 0.15	0.70 ± 0.52	0.7 ± 0.1	0.6 ± 0.3	0.9 ± 0.4	0.7 ± 0.2	0.8 ± 0.8
Albumin	3.9 ± 0.5	4.1 ± 0.4	3.9 ± 0.5	3.6 ± 0.6	3.7 ± 0.6	3.9 ± 0.14	2.4 ± 2.26	4.1 ± 0.2	3.7 ± 0.30	3.4 ± 0.51	4.1 ± 0.1	3.9 ± 0.4	3.8 ± 0.6	4.1 ± 0.2	3.9 ± 0.6
HgbA1c	5.8 ± 1.1	5.5 ± 0.6	5.8 ± 1.1	5.9 ± 1.2	6.6 ± 1.6	6.7 ± 1.6	―	6.5 ± 1.4	5.3 ± 0.2	5.6 ± 0.74	5.6 ± 0	6.3 ± 2.0	5.4 ± 0.4	5.1 ± 0.1	5.5 ± 0.8

Abbreviations: AI/AN, American Indian/Alaskan Native; BMI, body mass index; Decomp., decompensated liver disease; GT, genotype; HBV, hepatitis B virus; TE, treatment experienced.

^a^By liver biopsy, imaging (computed tomography, magnetic resonance imaging, ultrasound), Fibrosure, FIB-4, and/or APRI.

^b^F3-F4.

^c^Unknown subtype.

### Virologic Response to DAA Therapy

#### HCV GT1

**Figure 1.**
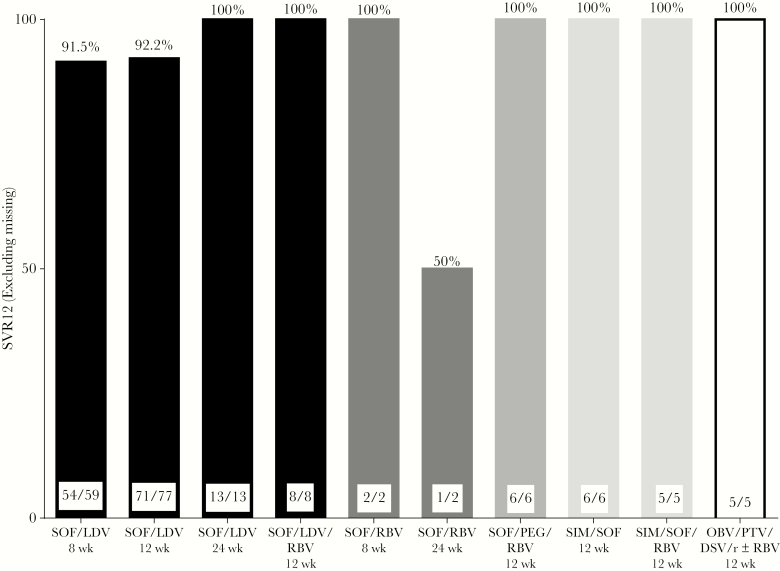
High rates of sustained virologic response at 12 weeks in American Indian/Alaska Natives patients with hepatitis C virus genotype 1. Abbreviations: DSV, dasabuvir; LDV, ledipasvir; OBV, ombitasvir; PEG, pegylated interferon; PTV, paritaprevir; RBV, ribavirin; SIM, simeprevir; SOF, sofosbuvir; SVR12, sustained virologic response at 12 weeks.

**Figure 2.**
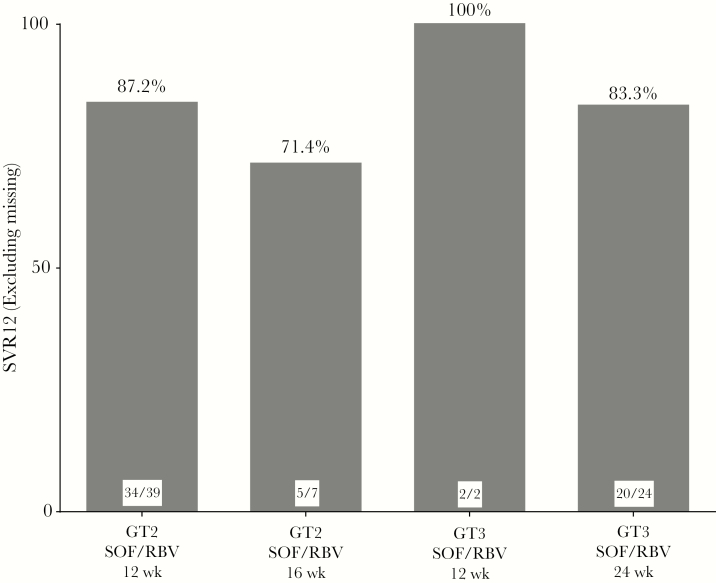
High rates of sustained virologic response at 12 weeks in American Indian/Alaska Natives with hepatitis C virus genotype 2 and genotype 3. Abbreviations: RBV, ribavirin; SOF, sofosbuvir; SVR12, sustained virologic response at 12 weeks.

**Figure 3.**
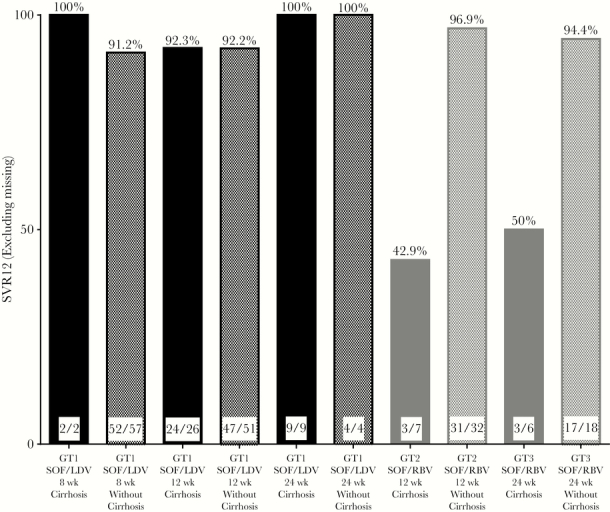
Rates of sustained virologic response at 12 weeks in hepatitis C virus genotype 1, genotype 2, and genotype 3 patients with and without cirrhosis. Abbreviations: LDV, ledipasvir; RBV, ribavirin; SOF, sofosbuvir; SVR12, sustained virologic response at 12 weeks.

Among the 199 HCV GT1 patients, 26.6% (n = 53) had cirrhosis, 13.6% (n = 27) were treatment experienced, and 85.4% (n = 170) had a baseline HCV RNA <6 million international units/milliliter (IU/mL). One-hundred sixty-five (83%) of these patients were treated with SOF/LDV, including 23.6% (n = 39) with cirrhosis. Overall, among patients treated with 8, 12, and 24 weeks of SOF/LDV, SVR12 was achieved by 91.5% (n = 54), 92.2% (n = 71), and 100% (n = 13), respectively (Figure 1). Rates of SVR12 were equally as high among patients with cirrhosis, with those treated for 8, 12, and 24 weeks achieving 100% (n = 2), 92.3% (n = 24), and 100% (n = 9) SVR12, respectively (Figures 2 and 3; Supplementary Tables 1 and 2).

Among the 34 GT1 patients treated with SOF/LDV/RBV, SOF/RBV, SOF/PEG/RBV, SIM/SOF, SIM/SOF/RBV, and OBV/PTV/DSV ± RBV, 97.1% (n = 33) of patients achieved SVR12 (Figure 1). The 1 patient who relapsed was treatment naïve but had cirrhosis and received 24 weeks of SOF/RBV.

Overall, among the 183 (92%) GT1 patients with SVR12 data, 11 (6.0%) patients relapsed. Five of these patients were treated with SOF/LDV for 8 weeks, all of whom were treatment naïve and noncirrhotic with baseline HCV RNAs <6 million IU/mL (1 patient was missing baseline HCV RNA data). Six GT1 relapse patients were treated with SOF/LDV for 12 weeks: 1 patient was treatment experienced with cirrhosis, and 1 patient was treatment naïve with cirrhosis; the remaining 4 were both treatment naïve and noncirrhotic.

### HCV GT2

Among 51 patients with HCV GT2, 21% (n = 11) had cirrhosis and 13.7% (n = 7) were treatment experienced. Forty-four patients were treated with SOF/RBV for 12 weeks, 87.2% of whom (n = 34) achieved SVR12. Of the 5 patients who relapsed on this regimen, 80% (n = 4) had cirrhosis. Forty-three percent of cirrhotic patients achieved SVR12.

Seventy-one percent (n = 5) of GT2 patients treated with SOF/RBV for 16 weeks achieved SVR12. Two patients relapsed after treatment, both of whom had cirrhosis (Figures 2 and 3; Supplementary Tables 1 and 2).

### HCV GT3

Thirty patients had HCV GT3, 30% (n = 9) of whom had cirrhosis and 6.7% (n = 2) of whom were treatment experienced. Among the 28 patients treated with 24 weeks of SOF/RBV, 83.3% (n = 20) achieved SVR12. Four HCV GT3 patients relapsed, of whom 75% (n = 3) had cirrhosis. SVR12 data were missing for 4 patients. Fifty percent of patients with cirrhosis treated with 24 weeks of SOF/RBV achieved SVR12. All patients (n = 2) treated with 12 weeks of SOF/RBV achieved SVR12 (Figures 2 and 3; Supplementary Tables 1 and 2).

### HCV Risk Factors

In the subset of 249 patients on whom additional HCV risk factor data were available, 96% (n = 240) of patients had at least 1 reported risk factor for acquiring HCV. Seventy-three percent (n = 181) of patients reported a history of intravenous drug use (IDU) (Table 2). Sixty-two percent (n = 155) of patients had tattoos, and 18.5% (n = 46) had a history of blood transfusions before 1992. Thirty-one percent (n = 76) of patients reported a history of sexual intercourse with a person who identified themselves as being infected with HCV.

**Table 2.** HCV Risk Factors, Comorbidities, and Immunity to Hepatitis A and B Among 249 AI/AN Patients

HCV Risk Factors	No. (%)
History of IVDU	181 (72.7)
Nonprofessional tattoos	155 (62.2)
Sex with HCV (+) partner	76 (30.5)
Current active substance use (UDS)^a^	50 (29.9)
Receipt of blood transfusion or blood products before 1992	46 (18.5)
Health care worker	39 (15.7)
Mother with HCV	21 (8.4)
Sharing personal items (ie, razors)	13 (5.2)
Current active substance use (IVDU)^b^	11 (4.4)
Comorbidities	
Alcohol use disorder	94 (37.8)
Depression	43 (17.3)
Anxiety	3 (1.2)
Other psychiatric disease	22 (8.8)
Type II diabetes mellitus	50 (20.1)
NAFLD^c^	33 (13.3)
ALD	14 (5.6)
History of tobacco use	173 (69.5)

Abbreviations: AI/AN, American Indian/Alaskan Native; ALD, alcoholic liver disease; HCV, hepatitis C virus; IVDU, intravenous drug use; NAFLD, nonalcoholic fatty liver disease; UDS, urine drug screen.

^a^One hundred sixty-seven patients had a UDS performed. Substances including cannabis, opioids, benzodiazepines, and amphetamines were being used by patients during HCV treatment without a prescription.

^b^Two hundred forty-eight patients received a manual chart review to verify whether they were actively injecting intravenous drugs while on treatment.

^c^Two hundred eleven patients had an abdominal ultrasound performed to screen for steatosis and other hepatic complications.

Approximately one-quarter of patients had a diagnosed psychiatric disease at the time of HCV evaluation (Table 2).

### NAFLD

Among patients who had a pretreatment ultrasound performed (n = 211), 22.3% (n = 47) showed evidence of steatosis. Thirty-three (70.2%) of these patients had steatosis due to NAFLD (ie, no history of alcohol abuse), whereas 14 patients with steatosis (29.8%) had a history of alcohol abuse that may have contributed to steatosis findings. There was no statistically significant difference in the prevalence of NAFLD across HCV genotypes (17.9% GT1, 10% GT2, 11.5% GT3, *P* = .39). In comparing HCV patients with and without concomitant NAFLD, there was no statistically significant difference in the prevalence of cirrhosis: 18.2% (n = 6) and 31.9% (n = 43), respectively (*P* = .46).

In regards to virologic response to HCV therapy, rates of SVR12 were similar in HCV patients with and without NAFLD (GT1: 95.8% vs 95.2%, *P* = .9057; GT2: 66.7% vs 95.5%, *P* = .2300; GT3: 100% vs 75%, *P* = 1.00, respectively).

### HCV Treatment Outcomes in Patients With Active Substance Use Disorder

During HCV treatment, 22.6% (n = 56) of eligible patients had active substance use disorder. Of these, 50 patients (89.3%) had a positive UDS for substances that were not prescribed, including cannabis, amphetamines, opiates, benzodiazepines, and barbiturates. Six (10.7%) patients were self-reported intravenous drug users (IVDUs) who either did not have a UDS performed (n = 4) or tested negative (n = 2).

Given the smaller number of patients with genotypes 2 and 3, the effect of active drug use on SVR12 was evaluated in patients with HCV GT-1 only. In this group, there were no differences in SVR12 between patients with and without active substance use disorder (GT1: 91.2% vs 94.5%, *P* = .4374.

### Patients Missing SVR12 Data and Patients who Relapsed

In the aggregate population, 25 (8.9%) patients were missing SVR12 data. Thirty-two percent (n = 8) had active substance use disorder due to cannabis, amphetamines, opioids, or barbiturates, among whom 50% (n = 4) were using intravenous drugs. Twenty-four percent (n = 6) of patients missing SVR12 data had some form of psychiatric disease, and 20% (n = 5) had cirrhosis. Neither age, race, active substance use disorder, psychiatric disease, nor cirrhosis (*P *> .05 for each comparison) was predictive of missing SVR12.

Overall, there were 23 patients who relapsed, of whom 52.2% (n = 12) had cirrhosis and 13% (n = 3) were treatment experienced. Again, neither alcohol use, NAFLD, cirrhosis, nor treatment experience was a predictor of relapse for patients with HCV GT1 (*P* > .05 for all).

## CONCLUSIONS

This study demonstrates that DAA therapeutic regimens are highly effective among HCV-infected AI/ANs. As a whole, SVR12 rates were comparable to the results observed in clinical trials, which disproportionately enroll Caucasian patients. Significantly, SVR12 rates remained high among patients with host and/or risk factors that typically undermine treatment effectiveness, including cirrhosis or thought to be undermine effectiveness, ie active substance use disorder.

Among GT1 patients treated with SOF/LDV for 8, 12, and 24 weeks, SVR12 rates were similar to those seen in other real-world effectiveness trials [12, 13]. No predictors of treatment failure for HCV GT1 were identified. Relative to clinical trials, GT2 patients with cirrhosis treated with 12 or 16 weeks of SOF/RBV demonstrated lower rates of SVR12 [19, 20]. Among the 6 GT2 patients with cirrhosis who relapsed, 50% (n = 3) showed signs of decompensation including esophageal variceal bleeding and hepatic encephalopathy, which may explain the lower rates of SVR12 seen in this cohort. Conversely, GT2 patients without cirrhosis showed SVR12 rates similar to those seen in other real-world effectiveness trials of SOF/RBV [19–21].

The prevalence of concomitant NAFLD (13.3%) was modest and likely underestimates the true prevalence given that the indication for ultrasound was hepatitis C and not evaluation for hepatic steatosis [22, 23]. NAFLD was not predictive of treatment failure; however, the sample size was small, with only 65.2% (n = 15) of relapsers having had an ultrasound performed. Additional studies are needed to characterize the impact of concomitant NAFLD/Non-Alcoholic Steatohepatitis in AI/AN patients with HCV [24, 25].

Similar to the US population as a whole, IDU among AI/ANs is the principal cause of HCV acquisition [26, 27]. Although treatment of patients with active substance use disorder would undoubtedly confer many benefits to the individual patient and on a larger public health scale, the practical application of DAAs remains controversial, as demonstrated by insurance policies in some states, restricting DAA treatment in patients who are actively using drugs. In this study, GT1 patients with active substance use disorder maintained high rates of SVR12. This high rate of SVR12 demonstrated in those actively using drugs has been seen in other studies of active drug users, and underscores the importance and effectiveness of treating HCV in all patients who are willing to be treated [28, 29] Increasing DAA treatment availability and the use of harm reduction interventions for patients with active substance use disorder will not only reduce prevalence but also make HCV elimination realistic [30–32].

The primary limitation of this study lies in its retrospective design. Additionally, small numbers of HCV GT2 and GT3 patients were included, particularly those with cirrhosis. Although our low rates of SVR12 among GT2 and GT3 patients with cirrhosis are concerning, these data should be re-evaluated with larger populations of AI/ANs and newer regimens.

Approximately 2.2 million AI/ANs receive their health care through Indian Health Services (IHS) [33]. With the expansion to universal HCV testing, IHS has taken important steps toward diagnosing HCV in a large at-risk population [34, 35]. Requests for medications still often have to go through Medicaid, Medicare, private insurers, or patient assistance programs. Unfortunately, many of these entities impose barriers in the approval process of DAAs, such as sobriety and fibrosis stage requirements. As such, more measures must be taken to eliminate these barriers and increase the provision of specialized care for this population of patients.

In summary, this study shows that DAA therapy is effective in AI/AN patients. As such, the use of DAAs for this often neglected population with disproportionate liver morbidity and mortality should be encouraged and expanded. As a whole, active substance use disorder should not be a barrier to HCV treatment and, in fact, is an added impetus to treat given the benefits to the individual patient and the community at large.

## Supplementary Data

Supplementary materials are available at *Open Forum Infectious Diseases* online. Consisting of data provided by the authors to benefit the reader, the posted materials are not copyedited and are the sole responsibility of the authors, so questions or comments should be addressed to the corresponding author.

ofz128_suppl_supplementary_table-1

ofz128_suppl_supplementary_table-2
