# Supplementary material for: Retrospective Study Demonstrating High Rates of Sustained Virologic Response After Treatment With Direct-Acting Antivirals Among American Indian/Alaskan Natives
Source: Open Forum Infect Dis. 2019 Jul 4;6(7):ofz128. doi: 10.1093/ofid/ofz128 (PMC6610205; doi:10.1093/ofid/ofz128)
Supplement: ofz128_suppl_supplementary_table-1 [file ofz128_suppl_supplementary_table-1.docx]

**Supplemental Table 1: Virologic Response to DAA Therapy Among 280 HCV-Infected AI/ANs**

|  | **GT1** | | | | | | | | | | **GT2** | | **GT3** | |
| --- | --- | --- | --- | --- | --- | --- | --- | --- | --- | --- | --- | --- | --- | --- |
|  | **SOF/LDV** | | | **SOF/LDV/RBV** | **SOF/RBV** | | **SOF/PEG/RBV** | **SIM/SOF** | **SIM/SOF/RBV** | **OBV/PTV/DSV/r ± RBV** | **SOF/RBV** | | **SOF/RBV** | |
| **Duration (Wks.)** | 8 | 12 | 24 | 12 | 8 | 24 | 12 | 12 | 12 | 12 | 12 | 16 | 12 | 24 |
| **N** | 69 | 83 | 13 | 8 | 2 | 2 | 6 | 6 | 5 | 5 | 44 | 7 | 2 | 28 |
|  | | | | | | | | | | | | | | |
| **SVR-12** | 54 (79.4) | 71 (85.5) | 13 (100) | 8 (100) | 2 (100) | 1 (50) | 6 (100) | 6 (100) | 5 (100) | 5 (100) | 34 (77.3) | 5 (71.4) | 2 (100) | 20 (71.4) |
| Missing (n=25) | 10 (40) | 6 (24) | ― | ― | ― | ― | ― | ― | ― | ― | 5 (20) | ― | ― | 4 (16) |
| **SVR-12 (missing excluded)** | **54 (91.5)** | **71 (92.2)** | **13 (100)** | **8 (100)** | **2 (100)** | **1 (50)** | **6 (100)** | **6 (100)** | **5 (100)** | **5 (100)** | **34 (87.2)** | **5 (71.4)** | **2 (100)** | **20 (83.3)** |
|  | | | | | | | | | | | | | | |
| **Relapsers** (n=23) | 5 (21.7) | 6 (26.1) | ― | ― | ― | 1 (4.3) | ― | ― | ― | ― | 5 (21.7) | 2 (8.7) | ― | 4 (17.4) |
| Cirrhosis (n=12) | ― | 2 (16.7) | ― | ― | ― | 1 (8.3) | ― | ― | ― | ― | 4 (33.3) | 2 (16.7) | ― | 3 (25) |
| TE (n=3) | ― | 2 (66.7) | ― | ― | ― | ― | ― | ― | ― | ― | ― | ― | ― | 1 (33.3) |
| Cirrhosis + TE (n=2) | ― | 1 (50) | ― | ― | ― | ― | ― | ― | ― | ― | ― | ― | ― | 1 (50) |

Abbreviations: treatment experienced (TE)
