# Supplementary material for: Retrospective Study Demonstrating High Rates of Sustained Virologic Response After Treatment With Direct-Acting Antivirals Among American Indian/Alaskan Natives
Source: Open Forum Infect Dis. 2019 Jul 4;6(7):ofz128. doi: 10.1093/ofid/ofz128 (PMC6610205; doi:10.1093/ofid/ofz128)
Supplement: ofz128_suppl_supplementary_table-2 [file ofz128_suppl_supplementary_table-2.docx]

**Supplemental Table 2: Virologic Response Among HCV-Infected AI/AN Patients With and Without Cirrhosis**

|  | **GT1** | | | | | | **GT2** | | **GT3** | |
| --- | --- | --- | --- | --- | --- | --- | --- | --- | --- | --- |
|  | **SOF/LDV** | | | | | | **SOF/RBV** | | **SOF/RBV** | |
| **Duration (Wks.)** | **8** | | **12** | | **24** | | **12** | | **24** | |
| **Cirrhosis** | **C** | **NC** | **C** | **NC** | **C** | **NC** | **C** | **NC** | **C** | **NC** |
| **N** | 2 | 67 | 28 | 55 | 9 | 4 | 7 | 37 | 9 | 19 |
|  | | | | | | | | | | |
| **SVR-12** | 2 (100) | 52 (77.6) | 24 (85.7) | 47 (85.5) | 9 (100) | 4 (100) | 3 (42.9) | 31 (83.8) | 3 (33.3) | 17 (89.5) |
| **Missing** | − | 10 (14.9) | 2 (7.1) | 4 (7.3) | − | − | − | 5 (13.5) | 3 (33.3) | 1 (5.3) |
| **SVR-12 (missing excluded)** | **2 (100)** | **52 (91.2)** | **24 (92.3)** | **47 (92.2)** | **9 (100)** | **4 (100)** | **3 (42.9)** | **31 (96.9)** | **3 (50)** | **17 (94.4)** |
|  | | | | | | | | | | |
| **Relapsers** | − | 5 (7.5) | 2 (7.1) | 4 (7.3) | − | − | 4 (57.1) | 1 (2.7) | 3 (33.3) | 1 (5.3) |
| TE | − | − | 1 (50) | 1 (25) | − | − | − | − | 1 (33.3) | − |

Abbreviations: Patients with Cirrhosis (C), Patients without Cirrhosis (NC), treatment experienced (TE)

Regimens with >10 patients are included.
